# Clinical effect of vein of Marshall ethanol infusion on mitral isthmus ablation

**DOI:** 10.3389/fcvm.2024.1253554

**Published:** 2024-02-05

**Authors:** Wei-Li Ge, Yi-Fei Lu, Tao Li, Ye Wang, Jie Yin, Xin-Ran Li, Jian-Jun Jiang, Ya-Fei Mi, Tao-Hsin Tung, Su-Hua Yan

**Affiliations:** ^1^ Shandong Provincial Qianfoshan Hospital, Shandong University, Jinan, Shandong, China; ^2^ Department of Cardiology, Taizhou Hospital of Zhejiang Province Affiliated to Wenzhou Medical University, Linhai, Zhejiang, China; ^3^ Department of Cardiology, Shandong Provincial Qianfoshan Hospital, Jinan, China; ^4^ Evidence-Based Medicine Center, Taizhou Hospital of Zhejiang Province, Wenzhou Medical University, Linhai, China; ^5^ Key Laboratory of Evidence-Based Radiology of Taizhou, Linhai, Zhejiang, China

**Keywords:** atrial fibrillation, catheter ablation, ethanol infusion, vein of Marshall, mitral isthmus (MI) ablation

## Abstract

**Purpose:**

This study aimed to investigate the effect of Marshall ethanol infusion (VOM-Et) in the vein on mitral isthmus (MI) ablation.

**Methods:**

Patients with persistent atrial fibrillation (AF) were grouped into vein of VOM-Et combined with radiofrequency (RF) ablation (VOM-Et-RF) and RF groups. The primary outcome was MI block immediate block rate after surgery. Stratified analysis was also performed for factors affecting the outcome measures.

**Results:**

A total of 118 consecutive patients underwent AF ablation at Taizhou Hospital of Zhejiang Province from January 2018 to December 2021. Successful bidirectional perimitral block was achieved in 96% of patients in VOM-Et-RF (69 of 72) and in 76% of patients in the RF group (35 of 46) (*P* < 0.01). In the subgroup analysis, male sex, elder than 60 years, Left atrial diameter <55 mm, and AF duration <3 years were associated with the benefits of VOM-Et in AF Patients.

**Conclusion:**

The vein of Marshall ethanol infusion for catheter ablation can improve the MI block rate. Male sex, elder age, smaller Left atrial diameter and shorter AF duration may have significant benefits for VOM-Et.

## Introduction

The prevalence of atrial fibrillation (AF) is increasing with the development of an aging society. At present, for catheter ablation of persistent atrial fibrillation, substrate modifications beyond pulmonary vein isolation is still lacking in evidence for further reducing the long-term recurrence. Some researchers believe that the long-term recurrence rate of persistent AF is related to mitral valve block rate (1).

Vein of Marshall ethanol infusion (VOM-Et) has become a popular topic in recent years. The block rate of the mitral isthmus (MI) can be improved by injecting absolute ethanol into the Marshall vein. In addition, VOM-Et can also play a role in intervening the autonomic nerve and AF triggers originating from the vein of Marshall, so as to reduce the recurrence rate of catheter ablation for persistent AF. However, few study has focus on the effect of VOM-Et on mitral isthmus ablation. The present study aims to investigate the impact of VOM-Et on MI ablation, as well as potential factors associated with the effect size.

## Methods

### Study design

**Figure 1:**
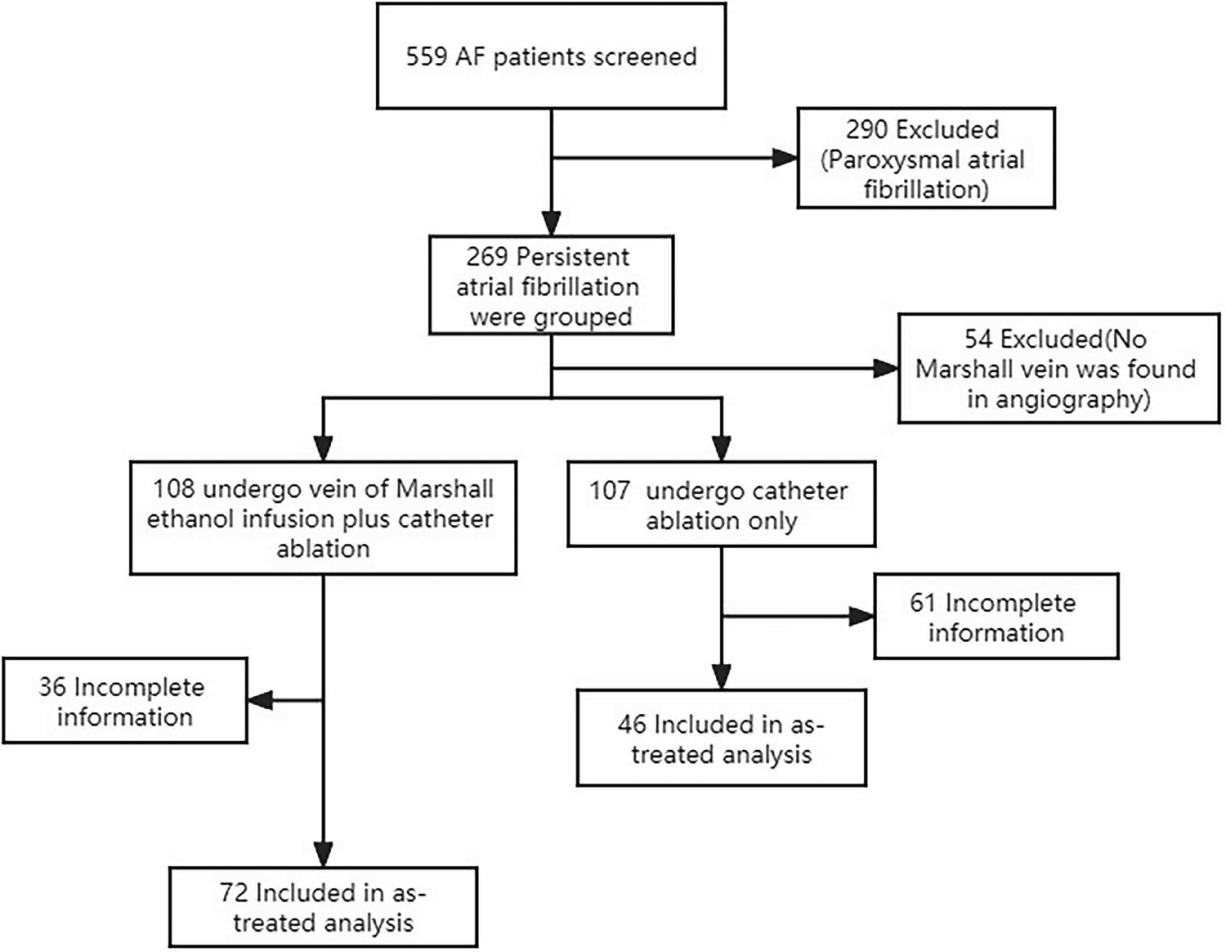
Study flow diagram of patient entrollment.

**Figure 2:**
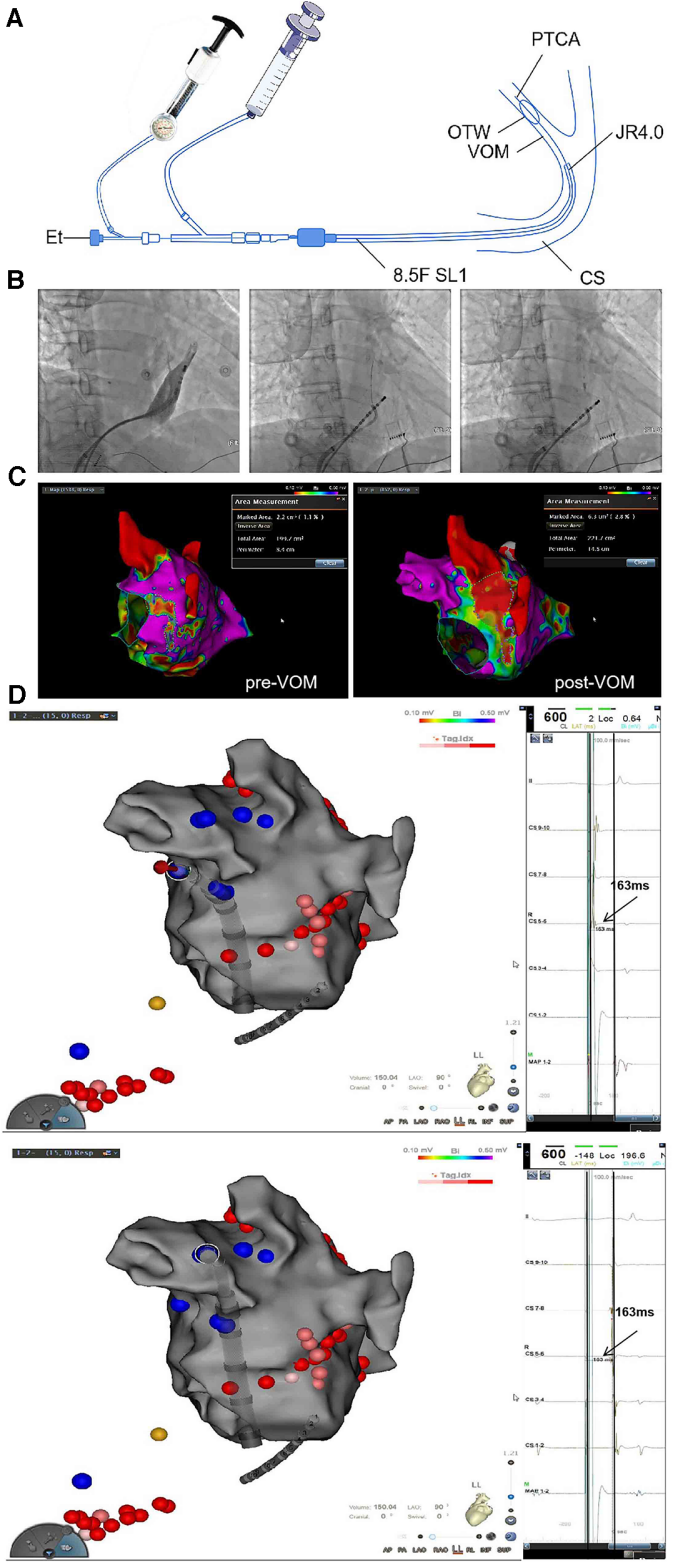
(**A**) A Schematic diagram of VOM-Et (1); (**B**) Selective angiography of the vein of Marshall; (**C**) Bi-polar voltage change in the mitral isthmus and left lateral ridge before and after EIVOM; (**D**) Verification of a bidirectional block of the mitral isthmus.

This is a single-center retrospective study which compared the effectiveness of rhythm control with two ablation strategies for persistent AF: RF alone or with Marshall vein ethanol ablation (VOM-Et-RF) (Figure 1). The protocol of the trial was reviewed and approved by the Institutional Ethics Committee. RF ablation applied a fixed anatomical ablation strategy including circumferential pulmonary vein isolation, left atrial roof, mitral isthmus, and tricuspid isthmus ablation, while additional VOM-Et was performed on the basis of aforementioned RF lesion sets in VOM-Et-RF group (Figures 2A–C).

In this study, the OTW balloon was fixedly placed at the proximal VOM. After inflation of the balloon at 6–8 atm, selective venography was performed to confirm total VOM occlusion, display the arborization of the VOM and identify collateral branches communicating with other structures. In cases, successive 5 times of ethanol was injected with 1–2 ml for each infusion and 1 min apart. At the end of VOM-Et, repeated venography was applied through the lumen of the balloon to observe tissue staining and exclude contrast extravasation into the free pericardium. After the ethanol fusion into the vein of Marshall procedure, we performed radiofrequency ablation.

### Setting and participants

A total of 118 consecutive patients underwent persistent AF ablation at Taizhou Hospital of Zhejiang Province from January 2018 to December 2021. Patients were eligible if they met the following inclusion criteria: (1) age 18–85 years; and (2) symptomatic persistent AF refractory to at least1 antiarrhythmic drug (AAD). The exclusion criteria were the following: (1) thrombus on transesophageal or intracardiac echocardiography; and (2) incomplete data.

All patients were divided into two groups according to ablation strategy, which were the following: (1) radiofrequency energy (RF) alone (*n* = 46); and (2) radiofrequency energy combined with vein of Marshall ethanol infusion (VOM-Et-RF) (*n* = 72).

### Variables

Patient anthropometric and procedural parameters were collected to analyze any significant association with mitral isthmus block (Figure 2D).

### Measurement

Clinical assessments were performed at baseline and after the initial ablation treatment under sinus rhythm, bidirectional block of MI was determined by differential pacing techniques (Figure 2).

### Quantitative variables

The correlation between MI block rate and alcohol ablation with sex, age, duration of atrial fibrillation, left atrial size, diabetes, hypertension, smoking, and alcohol consumption, was examined by correlation tests. Variables were identified with *P*-values <0.05 in univariate and multivariate analysis. Multivariate binary logistic regression analysis [odds ratio (OR) and 95% confidence interval (CI)] was performed to evaluate the predictors of MI block.

### Statistical methods

All continuous variables are presented as mean ± SD or median (range) if not distributed. Continuous data were compared using Student's *t*-test if normally distributed or Mann–Whitney *U*-test if not normally distributed. Categorical data were expressed as counts and proportions and compared using the *χ*^2^ test. Statistical threshold was set at *P* < 0.05. Statistical analysis was performed using the SPSS software (version 26.0; IBM Corporation, Somers, NY, USA).

## Results

### Baseline characteristics

The baseline characteristics of the patients are summarized in Table 1. A total of 118 patients were enrolled in this study between January 2018 and December 2021. The general patient characteristics were similar in all treatment arms. The mean patient age was 63.67 years. The majority of the study patients were male (73%), but the proportion of males in each group was similar. A repeat ablation procedure was performed in 46 (39.0%) of the RF group and in 72 (61.0%) of the VOM-Et-RF group. There were no significant differences in symptomatic AF episodes before inclusion in the registry between the two patient groups.

**Table 1:** Baseline characteristics.

Characteristic	RF (*n* = 46) [Number (%) or mean ± SD]	VOM-Et-RF (*n* = 72) [Number (%) or mean ± SD]	*P*-value for *χ*^2^-test or *t*-test
Age (y)	62.67 ± 8.7	64.31 ± 8.3	0.663
Male sex	30 (65.2)	48 (66.6)	0.513
Medical history and risk factors			
Hypertension	25 (54.3)	63 (87.5)	0.705
Diabetes	4 (8.7)	8 (11.1)	0.464
Coronary disease	1 (2.2)	2 (2.8)	0.664
Stroke/TIA	0 (0)	0 (0)	–
Heart failure	0 (0)	0 (0)	–
Body mass index (kg/m^2^)	24.93 ± 2.8	25.13 ± 3.4	0.054
CHA2DS2-VASc score	1.48 ± 1.07	1.65 ± 1.26	0.424
Cardiac parameters			
Ejection fraction (%)	58.52 ± 8.5	56.92 ± 8.0	0.643
Left atrial diameter (mm)	43.61 ± 5.6	43.64 ± 5.2	0.805
Time from first AF diagnosis			0.344
<3 year	23	43	
>3 year	23	29	

### Acute conduction block at the mitral isthmus

**Figure 3:**
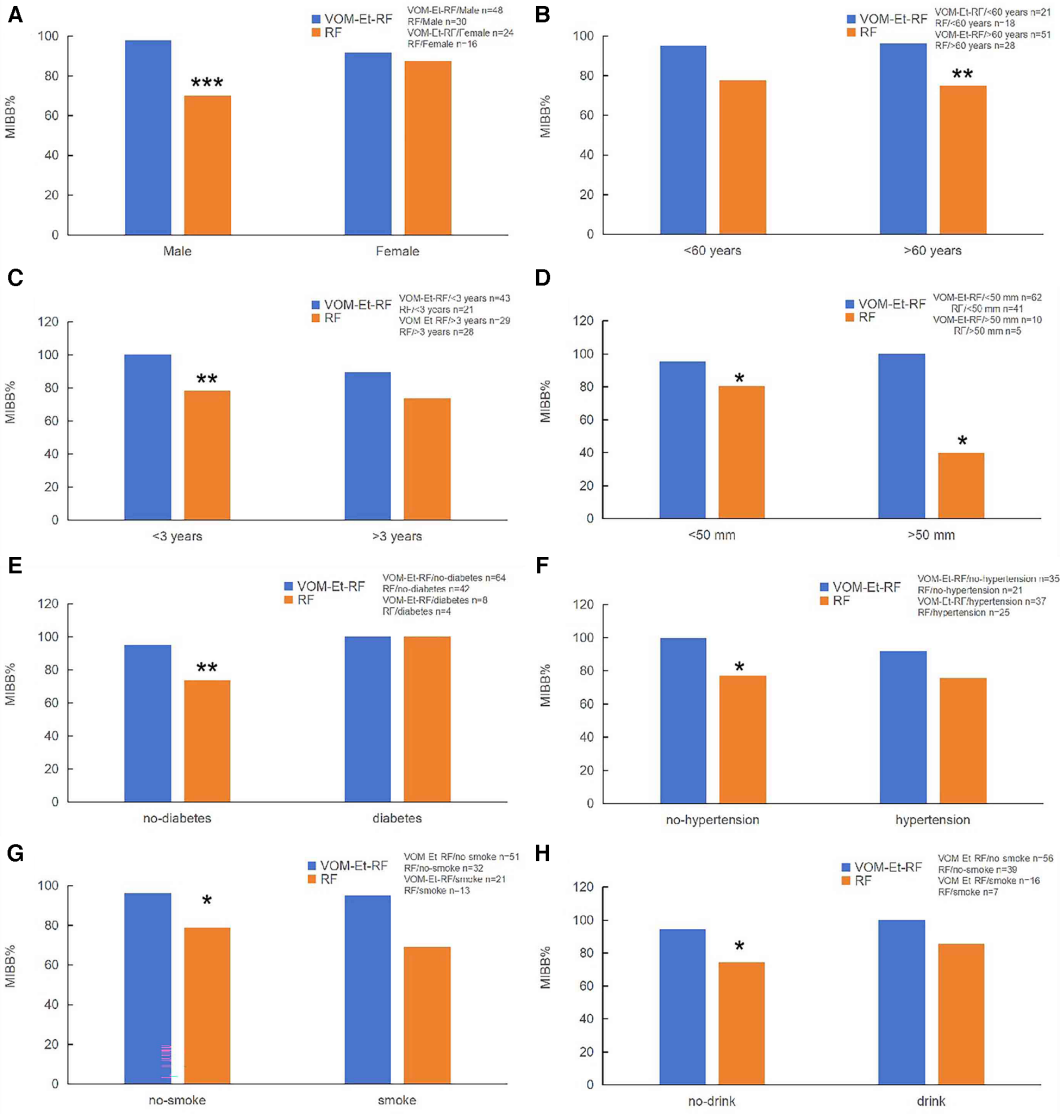
Influencing factor of acute conduction block at the MI. (**A**) Sex; (**B**) Age; (**C**) Duration of atrial fibrillation; (**D**) LA diameter; (**E**) Diabetes; (**F**) Hypertension; (**G**) Smoke; (**H**) Drink. VOM-Et-RF VS RF, *P < 0.05, **P < 0.01, ***P < 0.001.

Successful bidirectional perimitral block was achieved in 96% of patients in the VOM-Et-RF group (69 of 72) and in 76% of patients in the RF group (35 of 46) (*P* < 0.01) (Supplementary Figure S1). There is a significant intergroup difference in MI block rate for males (98% vs. 70%, *P* < 0.001), but no significant difference in female patients between the VOM-Et-RF group (91%) and RF group (86%) (Figure 3A). In terms of age, the rate of MI conduction block was significantly higher in VOM-Et-RF group (96%) than that in RF group (75%), in patients older than 60 years (Figure 3B).

For the analysis of the duration of atrial fibrillation, the rate of block at the MI was significantly higher in the VOM-Et-RF group (100%) than RF group (78.3%) in patients with duration of AF less than 3 years. However, there was no difference in the rate of perimitral block in patients with the duration of AF more than 3 years (Figure 3C). In addition, in patients with left atrial diameter less than 55 mm, the rate of isthmus block was significantly higher in the VOM-Et-RF group (95.2%) than in the RF group (80.48%), while in patients with left atrial diameter greater than 55 mm, the rate of isthmus block was significantly higher in the VOM-Et-RF group (100%) than in the RF group (40%) (Figure 3D).

Further analysis of clinicopathological factors affecting the progression of AF showed that in non-diabetic patients, the rate of perimitral block was significantly higher in the VOM-Et-RF group (95%) than in the RF group (73%), while there was no statistical difference in the contribution of the two groups to the rate of isthmus block in diabetic patients (Figure 3E). Similarly, in non-hypertensive patients, the rate of isthmus block was dramatically higher in the VOM-Et-RF group (100%) than in the RF group (77%), but there was no significant difference in the rate of perimitral block in hypertensive patients (Figure 3F). Similarly, in non-smoking and non-drinking patients, the rate of isthmus block was remarkably higher in the VOM-Et-RF group (98% and 96%, respectively) than in the RF group (78% and 74%, respectively), while there was no significant difference in the contribution of the two groups to the rate of isthmus block in the non-smoking and non-drinking patients (Figures 3G, H).

Our further stepwise regression analysis found that only LA diameter and whether alcohol ablation was performed or not were included in the regression curve, with a significant effect on MIBB%, and all the remaining variables were removed (Table 2).

**Table 2:** Analysis of the influencing factors of mitral isthmus block rate in patients.

	Univariate analysis			Multivariate analysis		
Variants	Odds ratio	95% CI	*P*-value	Odds ratio	95% CI	*P*-value
Gender	0.756	0.221–2.579	0.452			
Age	1.144	0.356–3.676	0.52			
Duration of atrial fibrillation	0.392	0.123–1.25	0.091			
LA diameter	0.058	0.005–0.691	0.037	0.057	0.004–0.892	0.041
Hypertension	0.577	0.181–1.840	0.259			
Diabetes	1.13	1.055–1.212	0.203			
Smoke	0.696	0.215–2.252	0.373			
Drink	3.488	0.432–28.133	0.193			
VOM-Et	7.229	1.893–27.601	0.002	1.581	1.581–28.785	0.01

## Discussion

The main findings of the present study, indicate that VOM ethanol infusion facilitates MI conduction block. Furthermore, in the subgroup analysis, our data support that male sex, age >60 years, LA diameter <55 mm and AF duration <3 years were independently associated with the benefits of VOM-Et for AF patients.

The success rates for achieving MI block using RF range from 42% to 92% for the posterior MI line (2–8), 85%–88% for the anterior MI line (9, 10), and 98.2% for the superolateral MI line (11). Acute and long-term success may be impacted by MI thickness and duration. A growing body of research has shown that VOM is significantly associated with arrhythmias, and the main potential pathophysiological mechanisms are proximal VOM autonomic nervous system imbalance, VOM-related AT, VOM-related reentrant activities triggering AF, and focal activities perpetuating AF (12). According to preliminary clinical studies, ethanol effusion into VOM can result in a complete linear lesion along the MI and successfully block the MI (13, 14), making it a viable treatment option for peri-mitral AT. In contrast to catheter ablation alone, catheter ablation with VOM-Et enhanced the likelihood of independence from AF/AT for PeAF with one-year follow-up, according to a recent randomized, multi-center experiment (VENUS trial) (1).

The difficulties in MI ablation and the low block rate is the major consideration that preclude application of MI ablation in clinical practice. Complete MI block may be difficult to achieve due to several physical restrictions, including a thicker and longer MI (15), the heat-sink effect of blood flow in the CS and circumflex arteries, and epicardial fibers like the CS myocardial sleeve and ligament of Marshall (16–18). These factors often result in longer ablation time and higher procedural risk. In our study, we found that the size of LA and the application of VOM-Et are key factors affecting the success rate of MI Block. Atrial structural remodeling is a common phenomenon in patients with atrial fibrillation (AF), usually manifested as increased atrial volume and atrial wall fibrosis, which will increase the success rate of catheter ablation to achieve MI block. Left atrial enlargement is closely related to the severity of atrial fibrillation, especially in persistent atrial fibrillation, which increases the success rate of both PVI isolation and MI block (19).With VOM-Et, MI ablation is much facilitated. Particularly, less RF applications are required in the mid to distal part of MI including the LAA base, which has more trabeculated and creviced structures susceptible to perforation. Although VOM rupture and leaking of ethanol may occur during VOM-Et and cause pericardial effusion or pericarditis, most of these complications could be manage with medical observation and appropriate medications.

It's also critical to understand VOM-Et's own limitations. The VOM is not always available, and the accessibility rate ranges from 71.4% to 96% (13, 20, 21). The difference in techniques, limited sample size of the reports could be the potential explanations that require further research. Of special note, the annulus side of MI is not covered by the VOM-Et lesion. As previously stated and anticipated due to anatomical factors, VOM-Et mostly affects the pulmonary venous side of the mitral isthmus while sparing the annular aspect (22). For mitral isthmus ablation, epicardial ablation via the CS is frequently necessary. Epicardial ablation often targets the annular side of the mitral isthmus due to the path of the CS, paying particular attention to the CS and GCV (23).

Although VOM-Et has a great help in improving the success rate of MI Block, there are some factors that can affect its effectiveness. Our study is the first to analyze individual factors that influence the clinical outcome of VOM-Et, including the sex, age, left atrial size, and duration of atrial fibrillation, as well as hypertension, diabetes, smoking, and alcohol consumption. These factors are also important for the success rate of radiofrequency catheter ablation for atrial fibrillation. In previous reports, it has been found that the success rate of catheter ablation of MI block is affected by the thickness of the isthmus, anatomic isomerism, and connection to the epicardium. It is well known that patients with atrial fibrillation will expand the atrium because of the long duration of AF and age, which will aggravate the degree of myocardial fibrosis. These conditions can lead to a thickening of the isthmus, resulting in a low success rate of MI block for traditional catheter ablation and the need for further VOM for complementary ablation. High blood pressure, diabetes, smoking and alcohol consumption are also risk factors for exacerbating the progression of atrial fibrillation, so it is not difficult to understand their influence on the effect of VOM-Et. Sex differences have been recognized in many aspects of AF management, including epidemiology, clinical presentation, and response to treatment (24). Although current studies reported trivial sex differences in sinus rhythm maintenance after ablation (25–27), there are clues indicating sex differences in the pathophysiology of AF, such as the prevalence of non-PV triggers, atrial fibrosis, PV reconnection rate, and thickness of epicardial adipose tissue (28–31). In the present study, we further suggested a lower MI block rate in male patients who tended to benefit more from VOM-Et.

Other clinically relevant outcomes, such as the AF burden rate of repeat surgeries, showed the benefits of Marshall ethanol as well technique complications were not increased by the VOM-Et technique. The overall aggressive ablation strategy adopted in both groups was consistent with adverse outcomes. These findings further confirm the safety and efficacy of VOM-Et in improving the success rate of MI block and optimizing AF catheter ablation

## Limitations

This study has several limitations. First, the study was a single-center, exploratory cross-sectional study, and the end point was immediate intraoperative MI block rate, mainly limited by the lack of follow-up and analysis of redo procedures, not possible to evaluate the long-term injury effect of VOM-Et and its contribution to the success rate of ablation of atrial fibrillation. Subgroup analysis should be interpreted with caution. Although we tried to enroll as many patients as possible, we still do not have adequate power to illustrate the effectiveness of VOM-Et in every subgroup. Therefore, a larger cohort with a longer follow-up period is warranted in the future.

## Conclusion

The present study provided further evidence for the effect of VOM-Et on MI ablation in the general population and different subgroups. VOM-Et is recommended to those more refractory to radiofrequency ablation, like younger, male patients.

## Data Availability

The original contributions presented in the study are included in the article/Supplementary Material, further inquiries can be directed to the corresponding authors.
