# Supplementary figures and images for: Clinical effect of vein of Marshall ethanol infusion on mitral isthmus ablation
Source: Front Cardiovasc Med. 2024 Feb 5;11:1253554. doi: 10.3389/fcvm.2024.1253554 (PMC10875083; doi:10.3389/fcvm.2024.1253554)

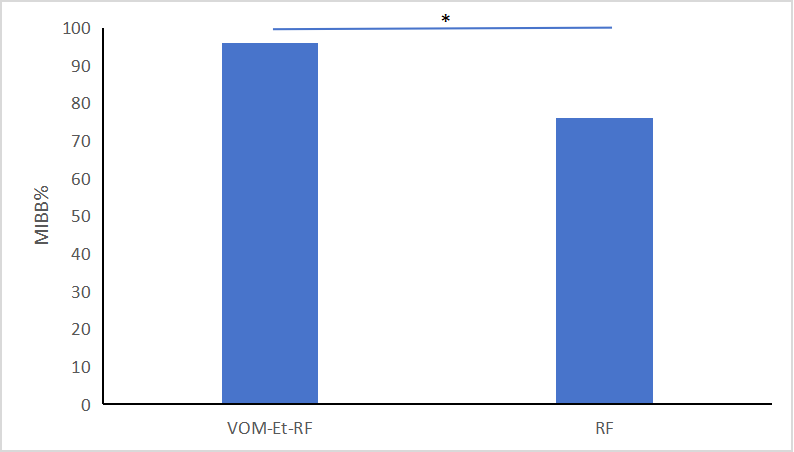

Supplement: Supplementary file 1 [file Image1.tif]
